# Seed Priming with Triacontanol Alleviates Lead Stress in *Phaseolus vulgaris* L. (Common Bean) through Improving Nutritional Orchestration and Morpho-Physiological Characteristics

**DOI:** 10.3390/plants12081672

**Published:** 2023-04-17

**Authors:** Shakil Ahmed, Minahil Amjad, Rehana Sardar, Manzer H. Siddiqui, Mohammad Irfan

**Affiliations:** 1 Institute of Botany, University of the Punjab, Lahore 54590, Pakistan; 2 Department of Botany and Microbiology, College of Science, King Saud University, Riyadh 11451, Saudi Arabia; 3 Plant Biology Section, School of Integrative Plant Science, Cornell University, Ithaca, NY 14850, USA

**Keywords:** growth, seed priming, triacontanol, lead stress, *Phaseolus vulgaris* L.

## Abstract

Worldwide, crop productivity is highly influenced by heavy metal toxicity. Lead (Pb) the is second-most toxic heavy metal that has high persistence in soil. Lead is translocated in plants from rhizosphere soil and enters the food chain, where it poses a significant hazard to the health of humans. In the present investigation, seed priming with triacontanol (Tria) was used to mitigate Pb phytotoxicity in *Phaseolus vulgaris* L. (common bean). Seeds were primed with different concentrations of Tria (control, 10 µmol L^−1^, 20 µmol L^−1^, 30 µmol L^−1^) solutions. The pot experiment was carried out by sowing Tria-primed seeds in contaminated soil with 400 mg kg^−1^ Pb. Lead alone induced a decrease in the rate of germination and a significant reduction in biomass and growth of *P. vulgaris* as compared to the control. All these negative effects were reversed by Tria-primed seeds. Proliferation of photosynthetic pigments was observed 1.8-fold by Tria under Pb stress. Primed seeds with 20 µmol L^−1^ Tria enhanced stomatal conductance (*gs*), photosynthetic rate (*A*), transpiration rate (*Ei*), and uptake of mineral contents (Mg^+2^, Zn^+2^, Na^+^, and K^+^) and reduced Pb accumulation in seedlings. Tria caused a 1.3-fold increase in osmotic regulator proline synthesis to alleviate Pb stress. Phenolics, soluble protein, and DPPH free radical scavenging activity were enhanced by Tria application, suggesting that exogenous Tria could be employed to improve plant tolerance to Pb stress.

## 1. Introduction

Heavy metals are one of the adverse chemicals that widely contaminate agricultural land and the environment due to their excessive release by industrialization and anthropogenic activities [1]. Some heavy metals (Fe, Mn, Cu, Zn, Mo) called micronutrients are essential to regulate metabolic processes of plants, whereas, As, Hg, Pb, Cr, and Cd can cause detrimental effects to growth and development of plants, as well as to animals if provided in minute concentrations [2]. Lead (Pb) is the second-most noxious heavy metal, which has no biological purpose. It is a hazardous metal with a high mobility from soil to plant, and thus has a particular impact on food quality and safety [3]. Lead is carcinogenic in nature, deteriorates soil fertility, and impedes soil microbial development, resulting in a reduction in agricultural productivity [4,5,6].

Lead causes phytotoxicity in plants, depending on time and concentration. Greater levels of lead exposure disrupt water and nutrient accumulation, as well as cause oxidative damage to plants [7]. Plant ontogeny, cell membrane permeability, chlorophyll content, photosynthesis, plant respiratory activities, cell division, and other biochemical and physiological processes have all been shown to be significantly hampered by Pb [5]. Furthermore, it causes the formation of a large number of reactive oxygen species (ROS), which disrupts the ultrastructure of cellular organelles, particularly cell membranes [8]. Plants may protect themselves from Pb stress in three ways: (a) by inhibiting Pb absorption by the roots (passive mechanisms), (b) by inducing an antioxidant network to combat ROS, and (c) by excreting Pb into the extracellular space or sequestering it in vacuoles (inducible mechanisms) [4,9]. The buildup of osmolytes substances such as proline, in addition to the antioxidant network, increases plant tolerance under stressful conditions by preserving osmotic potential and protecting cellular structures [10,11]. Elevated levels of Pb interfere in plants’ major metabolic and biochemical pathways, including photosynthesis, transpiration, respiration, antioxidative system, content of minerals, and even gene expression. Cell membrane permeability is also influenced by Pb phytotoxicity as it disrupts the lipid and protein composition of the membrane [12].

Seed priming is a physiological approach of seed hydration and drying to promote a pre-germinative metabolic process for quick germination, seedling development, and eventual yield under stress conditions [13]. When compared to unprimed seeds, primed seeds display quicker and more uniform seed germination due to differences in enzyme activation, metabolic activity, biochemical cell repair, protein synthesis, and enhancement of the antioxidant defense system [14]. Seed priming can reduce imbibition time, activating germination inducers, repairing DNA breaks, and managing moisture levels to ensure uniform germination [15]. Tria-primed seeds reduce heavy metal toxicity in plants and promote their development, growth, and productivity [16,17,18]. Salicylic acid (SA), brassinosteroids (BRs), jasmonic acids (JAs), triacantonol (Tria), auxin [19], gibberellic acid [20], and cytokinins [21], have all been reported as stress alleviators that improve tolerance to HM toxicity. Among them, Tria is a chemical classified as a phyto-protectant that belongs to saturated primary alcohols and cab be used as a seed primer. Triacontanol stimulates biochemical and physiological mechanisms of many crops, and it was recorded that at very low concentrations it enhances the growth of rice (*O. sativa* L.) and maize (*Zea mays* L.) [22]. In particular, Tria regulates gene expression when a plant is in stress conditions by increasing its enzymatic and non-enzymatic antioxidant defense systems. Notably, it also increases photosynthetic pigments and initiates growth along with increasing the compatible osmolyte accumulation in plants. [23]. Exogenous application of Tria boosts plant biomass, nutrient uptake, photosynthetic pigments, transpiration rate, soluble sugars, proteins, free amino acids, nitrogen fixation, crop yield, essential oil, and active constituents of plants [24,25].

*Phaseolus vulgaris* L. (common bean) is a vital grain legume cultivated around the world, mainly in developing countries. Common beans provide multiple health benefits and are a great source of protein, starch, dietary fiber, vitamins, (thiamine, vitamin B6), folic acid, and minerals, and offer numerous health benefits to consumers [26]. According to a survey conducted in 2019, the common bean was cultivated on 33,066,183 ha land in the world and a yield of 874.0 kg/ha was attained [27]. Due to its vast benefits to humans, this pulse crop is widely appreciated and used in developing countries. The common bean is important because of its affordable and high protein content comparable to animal protein, and also important in countries with mineral deficiencies due to its long storage life [28]. Lead has adverse effects on *P. vulgaris* growth and inhibits biochemical reactions, leading to a reduction in chlorophyll content and enhancing the proline content [29]. On the other hand, Pb reduces the uptake of macro and micro elements in common beans and shows a decline in plant growth, flower development, grain development, seed germination, and common bean yield under stress of Pb [26].

Various studies have mentioned the beneficial effects of various plant growth regulators on the growth and production of field crops. However, there is very little information about seed priming with Tria in vegetable crops. Moreover, there is almost no information about the role of Tria on growth, biomass, and nutrient content of *P. vulgaris* under Pb stress. Therefore, the goal of the current study was to determine the benefits of seed priming with Tria on agronomic attributes, physiochemical traits, and nutrients content of *P. vulgaris* subjected to Pb stress.

## 2. Materials and Methods

### 2.1. Seeds Procurement and Priming

Viable seeds of common beans (*Phaseolus vulgaris* L.) were purchased from Rasheed Seed Corporation, Gujranwala, Pakistan. Seeds were sterilized for 5 min by submerging in sodium hypochlorite (5%). After that, seeds were washed 3 times with the help of distilled water and subsequently air-dried by keeping them at 25 °C. Triacontanol (Tria) was acquired from Sigma Aldrich (Saint-Louis, MO, USA). To make the 1 mM Tria stock, 219.4 mg of the chemical was dissolved in 500 mL of distilled water. This stock solution was then used to prepare three concentrations, i.e., 10, 20, 30 µmol L^−1^ of Tria. Seeds were primed for 24 h under dim light at 25 ± 1 °C in varying concentrations of triacontanol (10 µmol L^−1^, 20 µmol L^−1^, 30 µmol L^−1^), regarded as Tria 1, Tria 2, and Tria 3, respectively. Following priming, seeds were washed thoroughly with distilled water and placed over blotting paper at room temperature for air-drying for 3 h under dim light at room temperature.

### 2.2. Pot Experimental Design

The experiment was executed in the Botanical Garden, University of the Punjab, Quaid-e-Azam Campus, Lahore (74°21-00-E, 31°35-00-N). For the experiment, four replicates of each treatment (Control, Pb, Tria 1, Tria 2, Tria 3, Tria 1 + Pb, Tria 2 + Pb, Tria 3 + Pb) were prepared by filling it with 2 kg of soil and amending with 400 mg kg^−1^ Pb with the help of lead acetate (PbC_4_H_6_O_4_). This was selected based on the potential risk of pollution in agricultural soil starting from 100 mg kg^−1^ of Pb for growing crops, and the Pb concentrations below the safe maximum allowable limits (MAL 350 mg kg^−1^) established by State Environmental-Protection Administration, China (SEPA), for soils in China. These limits are used as a reference as no such values have been established in Pakistan. Then, Randomized Complete Block Design (RCBD) was followed to arrange pots for the provision of similar environmental conditions. Initially, 5 primed and control seeds were sown in each pot at a distance of 2 inches and at 1 inch deep. Eleven-day-old seedlings were thinned and two seedlings were allowed to grow in every pot. After 41 days, the seedlings were uprooted, carefully washed with distilled water, and dried at 25 ± 1 °C for 10 min on blotting paper for their further physio-biochemical analysis.

### 2.3. Morphological Parameters

Forty-one days after sowing, morphological parameters and shoot and root length were measured, number of leaves was counted, and leaf area was formulated by using the Carleton and Foote method [30].

Leaf area = length of leaf × maximum leaf width × 0.75 (correction factor)

### 2.4. Biomass Assessment

Biomass parameters were assessed on the electric weighing balance, including the fresh weight of the shoots and roots. Next, these shoots and roots were air-dried for 24 h and placed in labelled paper bags for oven drying (Wise Ven, Model WOF-105, Republic of Korea) for complete drying. The temperature of the drying oven was set to 70 °C; plants were placed inside it for 72 h so that plants would become completely devoid of moisture. The dried shoots and roots were then weighed again using an electric weighing balance to assess the dry weights of shoots and roots (Sartorious GMBH, Type 1216MP 6E, Gottingen, Germany).

### 2.5. Photosynthetic Pigments Quantification

Photosynthetic pigments chlorophyll *a*, chlorophyll *b*, and total chlorophyll content by Arnon [31], and carotenoids by Davies [32], were calculated for all treated and control plants samples. First, a 0.5 g sample of fresh leaves was taken and crushed well by pestle and mortar. Then, 10 mL 80% acetone solution was added in each sample and left overnight in a cool, dry place. After that, the extracts were centrifuged at 1000 rpm for 5 min at 4 °C. Optical density of supernatants was then measured via a UV-spectrophotometer (UV-1800 Schimadzu) at 645, 663, and 480 nm and measured by the following formulas:

$$Chlorophyll\;‘a’\;\left(\frac{\mathrm{mg}}{g}\mathrm{FW}\right)=\left[\frac{\left(0.0127\times A663-0.00269\times A645\right)\times 100}{0.5}\right]$$

$$Chlorophyll\;‘b’\;\left(\frac{\mathrm{mg}}{g}\mathrm{FW}\right)=\left[\frac{\left(0.0229\times A645-0.00468\times A663\right)\times 100}{0.5}\right]$$

$$Total\;Chlorophyll\;\left(\frac{\mathrm{mg}}{g}\mathrm{FW}\right)=\left[\frac{\left(0.0202\times A645-0.00802\times A663\right)\times 100}{0.5}\right]$$

$$Carotenoids\;\left(\frac{\mathrm{mg}}{g}\mathrm{FW}\right)=\left[\frac{A480+0.114\left(A663\right)-0.638\times A645}{2500}\right]\times 1000$$

### 2.6. Estimation of Gas Exchange Attributes

The net photosynthesis rate (*A*), transpiration rate (*Ei*), and stomatal conductivity (*gs*) were analyzed at 12:00 p.m.–1:00 p.m. from the uppermost fully extended leaves of plants by using a portable Infra-Red Gas Exchange Analyzer (IRGA) (LCA-4 System ADC, Ltd.) [33].

### 2.7. Determination of Proline Content

Proline determination was conducted by the method described by Bates [34]. First, 0.25 g of plant sample was used and crushed by adding 10 mL of 3% sulpho-salicylic acid. The mixture was then centrifuged at 5000 rpm for 30 min. Then, 2 mL of supernatant was collected and 2 mL of acid ninhydrin (2.8 g ninhydrin, 48.16 mL of 85% Phosphoric acid and 72.8 mL acetic acid) and glacial acetic acid was added to it; the mixtures were vortexed and kept in a water bath (N.S Engineering concern XMTG-9000) for 1 h at 100 °C. After 1 h, the mixtures were added to an ice-chilled container to terminate the reaction. After cooling, 4 mL of toluene was vortexed for 30 s. Chromophore aspiration containing toluene was allowed to occur. Afterwards, the solution was placed at 25 °C for half an hour and optical density was obtained at 520 nm by using a (UV-1800 Schimadzu) spectrophotometer. Proline was determined by using a standard curve of L-Proline, and calculated on the basis of fresh weight as given below:

$$Proline\;\left(µ\frac{\mathrm{mol}}{g}\mathrm{FW}\right)=\left[\frac{µg\;\mathrm{proline}/\mathrm{mL}\;\times \;\mathrm{mL}\;\mathrm{of}\;\mathrm{toluene}\;}{115.5}\right]/\left[\frac{g\;\mathrm{of}\;\mathrm{sample})}{10}\right]$$

### 2.8. DPPH Free Radical Scavenging Activity Test

2,2 diphenyl-1-picryl-hydrazyl free radical scavenging activity of *Phaseolus vulgaris* was determined by the method described by Chen [35]. First, 1 g of plant sample was taken, and 20 mL of methanol was added to it to make methanolic extract. Then, 1ml of methanolic extract was taken and 5 mL of freshly prepared 0.1 mM DPPH methanolic solution was mixed thoroughly and left for 60 min in the dark for the reaction to proceed. Methanol (1 mL) was taken as blank, and optical density was obtained at 517 nm with a (UV-1800 Schimadzu) spectrophotometer. Percentage DPPH Scavenging Activity was calculated by following formula:

$$\mathrm{Scavenging Activity}(\%)=\left[1-\left(\frac{A517\mathrm{nm},Sample}{A517\mathrm{nm},Blank}\right)\right]\times 100$$

### 2.9. Estimation of Soluble Protein Content

For the estimation of protein in plants, the protocol by Peterson [36] was considered. First, 1 g of each treated and control leaf sample was crushed by using a pestle and mortar, using 2 mL 1N phosphate buffer (17 g K_2_HPO_4_ in 1000 mL of distilled water), and mixtures were then centrifuged at 6000 rpm for 15 min. After centrifugation, 0.4 mL of supernatant was taken and mixed with 2 mL of Folin mixture and left for 15 min. Then, 0.5 mL Folin’s Ciocalteau Reagent was added to each sample and placed at room temperature for 45 min after shaking. Optical density was then obtained at 715 nm by using a (UV-1800 Schimadzu) spectrophotometer. Soluble protein content was calculated by using a BSA (Bovine Serum Albumin) standard curve.

### 2.10. Lead Accumulation in Plants

To assess the amount of lead in plants, acid-digested samples were prepared by drying shoots at 65 °C for 48 h in an oven (Wiseven, Model WOF-105, Republic of Korea). After drying, samples were finely crushed in a pestle and mortar and passed through a 60-screen mesh. Then, 0.5 g of sample was obtained and 5 mL of 70% HNO_3_ and 1.5 m of 60% HClO_4_ were mixed to it. Next, the samples mixture was heated until the brown fumes disappeared. Then, the samples were cooled down at room temperature and 5 mL of diluted 50% HCl was added. These samples were then diluted by adding 25 mL of distilled water and carefully filtered [37]. An Atomic Adsorption Spectrophotometer (XPLOR AA-Dual) was used to determine the concentration of Pb in shoots, which was calculated by using the standard curve of Pb (Chapman, 1976). To find out the accumulation coefficient (AC), the ratio of concentration of Pb in plant by concentration of Pb in soil was calculated [38].

$$AC\;Factor=\frac{Concentration\left(Shoot\;and\;Root\right)}{Concentration\;of\;Soil}$$

In addition, the Metal Tolerance Index (*MTI*) was calculated by using the formula given by Balint [39].

$$\%MTI=\frac{Dry\;Weight\;of\;Treated\;Plants}{Dry\;Weight\;of\;Untreated\;Plants}\times 100$$

### 2.11. Mineral Contents Assessment

For assessing the concentration of minerals (Zn^+2^, Mg^+2^, Na^+^, and K^+^), the same acid-digested sample extracts were taken. Zn^+2^ and Mg^+2^ mineral concentrations were analyzed by using an Atomic Adsorption Spectrophotometer (XPLOR AA-Dual) and calculated by using the standard curves of Zn and Mg [40]. For the investigation of Na^+^ and K^+^ concentration, a Flame Photometer (Model 410, Corning) was used and calculated by using standard curves of Na and K [41].

### 2.12. Total Phenol Quantification

For sample preparation, 2 g of fresh leaves was taken and extracted by adding 10 mL of 80% aqueous methanol and heated at 65 °C for 15 min. Then, 1 mL of this methanolic extract, 250 µL of 1N Folin’s Ciocalteau reagent, and 5 mL of sterilized distilled water were homogenized by mixing and placed at 30 °C according to the method of Singleton and Rossi [42]. Absorbance of this blue-colored mixture was then observed at 725 nm by spectrophotometer (UV-1800 Schimadzu), and total phenol content was quantified by comparing the optical density value with the standard curve of gallic acid.

### 2.13. Statistical Analysis

Attained data were analyzed statistically by using IBM SPSS Statistics version 20 via applying one-way ANOVA, and the means were compared by applying Duncan’s Multiple Range Test (DMRT) observed at the significance level *p* ≤ 0.05. Two-way ANOVA and means were compared by applying Duncan’s Multiple Range Test (DMRT) observed at the significance level *p* ≤ 0.05. To quantify relationships between the numerous studied variables, Pearson’s Correlation Analysis was used. Rstudio was used to determine the Pearson correlation coefficients and Principal Component Analysis (PCA) between the *P. vulgaris* measured variables.

## 3. Results

### 3.1. Morphological Parameters

Table 1 shows the variation in the germination percentage and morphological parameters under the influence of Pb stress (Figure S1). It was observed that pots with Pb-stressed seedlings show a 17.6% lower germination percentage than control seedlings. The highest GP were noted at Tria-2 as compared to Tria-1 and Tria-3, both in control and Pb-contaminated treatments. The highest triacontanol germination was recorded at concentration 20 µM (Tria-2) and was 11.7% higher than control seedlings. Lead toxicity showed significant reduction in all growth parameters, although seed priming with triacontanol enhanced the growth traits both in control and Pb-amended soil.

Under lead regimes, there is a significant reduction observed in growth attributes comparative to control, i.e., shoot length, root length, no. of leaves, and leaf area by 33.1%, 33.6%, 23.5%, and 57.3%, respectively. However, when compared to lower concentrations of triacontanol with lead (10 µM and 20 µM), the values of these parameters decreased at the highest dose of triacontanol with lead (30 µM). The maximum increase in shoot length, root length, no. of leaves, and leaf area were noted in Tria-2 without lead (20 µM), with a 64.7%, 85.4%, 47%, and 63.5% increase, respectively.

Application of Tria had positive impacts on germination percentage (Table 2). A decreased germination rate percentage (17.6%) was observed in Pb stress without Tria treatment. Furthermore, Pb stress decreased growth attributes, but application of 20 µM Tria enhanced shoot length, root length, no. of leaves, and leaf area by 64.7%, 85.4%, 47%, and 63.5%, respectively, in control seedlings. The number of leaves decreased (67%) significantly in Pb-subjected seedlings over the control environment. Moreover, Tria treatment of Pb-subjected plants increased the shoot length and root length, but out of three treatments of Tria, 20 µM Tria triggered a maximum upsurge of 58%, which was 59% over control plants.

### 3.2. Biomass Assessment

Seedlings exposed to Pb stress had reduced fresh weights of shoot and root over control seedlings of about 62% and 41%, respectively. Conversely, it was noted that primed seeds had a positive effect on the fresh and dry biomass production. Lead-exposed seedlings decreased in dry shoot (45%) and root biomass (33%) production compared to control seedlings. It can be observed from Table 2 that as the concentration of Tria increased, there was an increase in shoot and root fresh and dry biomass, but at a higher dose of Tria, there was a slight decline both in the control and Pb-stressed plants. The application of Tria-2 enhanced the fresh weight of shoot (69%) and root (52%), and the dry weight of shoot (51%) and root (66%) over non-primed seedlings. Seed primed with Tria-3 showed a decrease in fresh and dry biomass production in both control and Pb-stressed conditions.

### 3.3. Photosynthetic Pigments Quantification

Positive impacts were observed in chlorophyll *a*, *b*, total chlorophyll, and in carotenoids in plants whose seeds were pre-treated with Tria both in Pb- and non-stressed soil conditions. Plants show a reduction in photosynthetic pigments in Pb-contaminated soil (Table 3). Pb reduced the chlorophyll *b* (0.58 mg/g FW) synthesis more than chlorophyll *a* (0.69 mg/g FW). A decline of 23% in Chl *a*, 15% in Chl *b*, 10% in total chlorophyll, and 18% in carotenoids production was observed, which results in reduced growth and development of plants when compared with control seedlings.

Triacontanol enhances the production of photosynthetic pigments, as maximum yields were shown in Tria-2 (20 µM), with an increase of about 62%, 86%, 63%, and 58% of Chl *a*, Chl *b*, total chlorophyll, and carotenoids, respectively. Tria also imparts positive effects on plants grown in Pb-stressed conditions by increasing photosynthetic pigments synthesis, with maximum yields in Tria-2 + Pb shown by an increase of 89% in Chl *a*, 86% in Chl *b*, 44% in total chlorophyll, and 40% in carotenoids, contrarily to Pb plants.

### 3.4. Estimation of Gas Exchange Parameters

Pb-stressed plants exhibit a noteworthy decline in gas exchange parameters, i.e., stomatal conductance (*gs*), transpiration (*Ei*), and net photosynthetic rate (*A*) in contrast to the control. Seeds primed with Tria, on the other hand, improved development characteristics and alleviated Pb stress in common beans.

**Figure 1:**
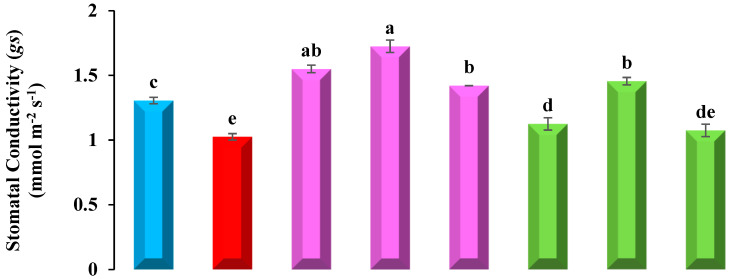

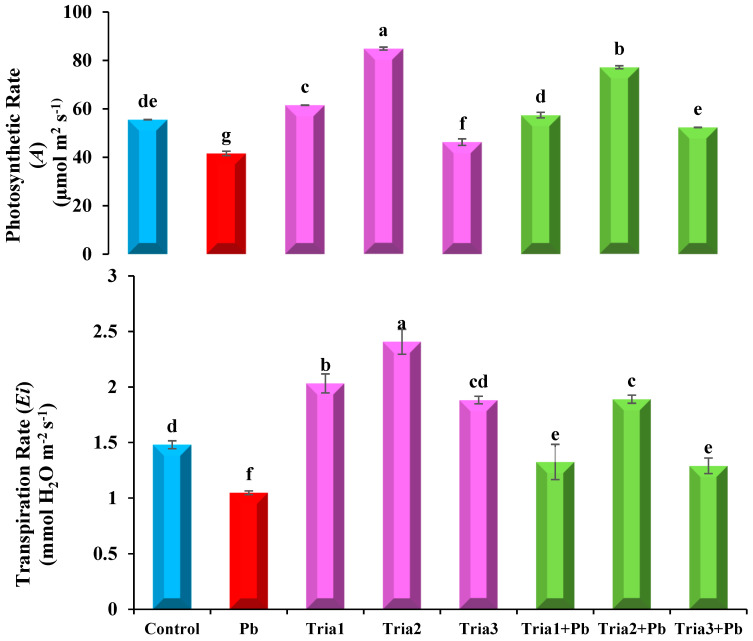
**Effect of Tria and Pb on Gas Exchange Attributes of *Phaseolus vulgaris* L.** (Data exhibit means ± SE of 4 replicates. Non-identical letters specify significant difference amid the treatments at *p* ≤ 0.05. Control, Pb = 400 mg kg^−1^ Pb, Tria-1 = 10 µmol L^−1^ of Tria, Tria-2 = 20 µmol L^−1^ of Tria, Tria-3 = 30 µmol L^−1^ of Tria.

Plants treated with Pb only showed a 21%, 25%, and 25% decrease in stomatal conductance (*gs*), transpiration (*Ei*), and net photosynthetic rate (*A*), respectively, than in control leaves. In seeds pre-treated with 20 µM Triacontanol Tria, shown in (Figure 1), Tria-2 shows maximum gas exchange attributes, i.e., a 24% increase in stomatal conductance (*gs*), 71% increase in transpiration (*Ei*), and 52% increase in net photosynthetic rate (*A*), when compared with control plants. Primed plants grown in Pb-stressed soil also shows significant results as Tria-2 + Pb shows a 42% increase in stomatal conductance (*gs*), 81% enhance in transpiration (*Ei*), and 85% increase in net photosynthetic rate (*A*), contrarily to solely lead-treated plants.

### 3.5. Determination of Proline Content

**Figure 2:**
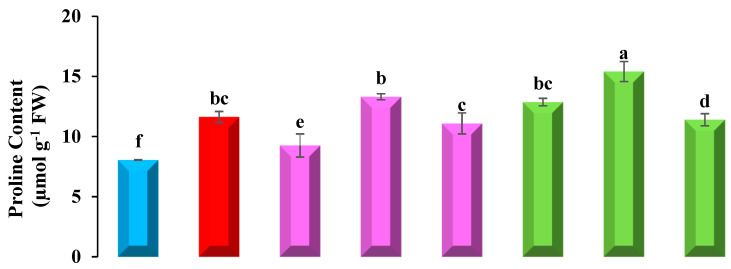

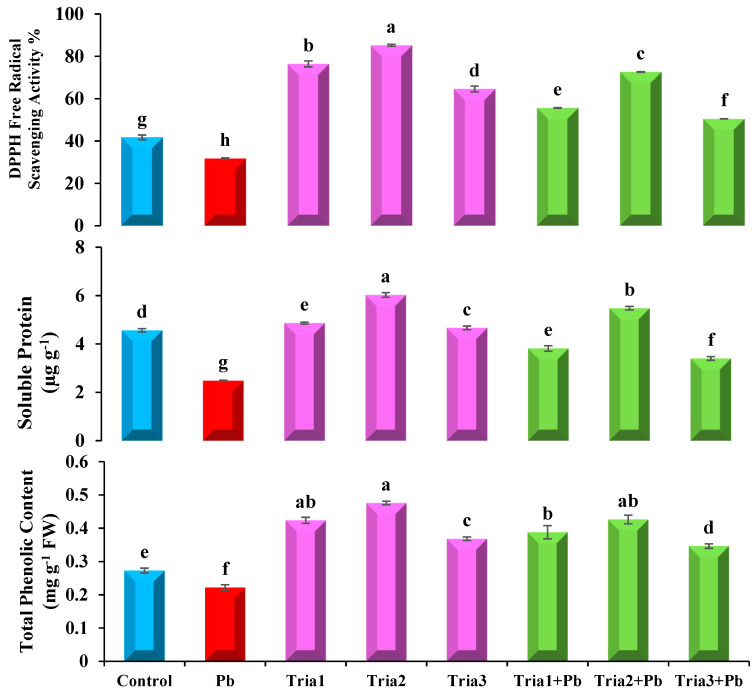
Effect of Tria on Proline Content, Antioxidant Activity, Total Soluble Protein and Total Phenolic Content of *Phaseolus vulgaris* L. under Pb stress. (Data exhibit means ± SE of 4 replicates. Non-identical letters specify significant difference amid the treatments at *p* ≤ 0.05. Control, Pb = 400 mg kg^−1^ Pb, Tria-1 = 10 µmol L^−1^ of Tria, Tria-2 = 20 µmol L^−1^ of Tria, Tria-3 = 30 µmol L^−1^ of Tria.

A higher level of proline synthesis was demonstrated by using an L-Proline standard curve in (Figure 2), for seedlings primed with Tria and grown in Pb-stressed soil, which indicates that in stress conditions, proline synthesis increases. A significant increase was recorded in only Pb-stressed plants, with an increase of about 44% in bean plant when compared with plants growing in control conditions.

In Tria-primed seeds at Tria-2 + Pb, a 91% increase in proline was revealed in Pb-stressed soil. In primed seeds without Pb stress, Tria-2 also enhanced the production of proline, but not more than in Pb-stressed soil (65%).

### 3.6. DPPH Free Radical Scavenging Activity Test

fPb-induced phytotoxicity significantly reduced DPPH radical scavenging activity, i.e., the percentage of free radical inhibition to predict the antioxidant activities. About a 23% decline was revealed in only Pb-supplemented plants compared to the control. An increase in stress reduces the scavenging activity of free radicals. Triacontanol reduces the Pb phyto-toxicity, and therefore enhances the scavenging activity of plants (Figure 2). Tria-2 (20 µM) showed a significant increase of about 2 folds in the control and 1.3 folds in Pb-amended soil. Plants grown in control conditions show more efficient results than plants in Pb settings.

Triacontanol also had a beneficial influence on DPPH radical scavenging activity, i.e., the percentage of free radical inhibition to predict antioxidant activities in common bean seedlings in both non-contaminated and Pb-stressed situations. Triacontanol inhibits Pb phytotoxicity and thereby increases plant scavenging activity (Figure 2). Exogenously applied Tria (Tria 1 and Tria 2) enhanced DPPH activity (49% and 64%, respectively) in contrast to control environments; however, Tria3 had no significant effect on DPPH content under Pb-stressed conditions.

### 3.7. Estimation of Soluble Protein Content

Pre-treatment of seeds with triacontanol Tria positively modulates the protein content of plants both in Pb and control soil, but in varying instants (Figure 2). Triacontanol at 20 µM shows the maximum result of about 6.02 µg/g, which is 1.3 times more than in the control, and in Pb-amended soil it shows a result of 5.4 µg/g, which is 2.2 times increased compared to only Pb. When Pb-only plants were compared with the control, they showed a drastic decline of 46% in their protein content. Pb inhibits the plant capability to produce protein, which ultimately reduces the growth parameters.

### 3.8. Lead Accumulation in Plants

Pb uptake by *P. vulgaris* was examined; the maximum in the Pb-spiked soil was about 0.17 mg g^−1^ DW, and the minimum was found in Tria-2 and was about 0.10 mg g^−1^ DW (Table 4). Supplementation of Tria also abridged the accumulation of Pb in plants grown under Pb regimes, and a similar decline was also documented in Tria-1 (34%), Tria-2 (41%), and Tria-3 (29%). Supplementation of Tria also abridged the accumulation of Pb in plants grown under Pb regimes, with a decline documented in Tria-1 (34%), Tria-2 (41%), and Tria-3 (29%). The metal tolerance index (MTI) shown in Table 4 was decreased by the Pb contamination and conversely increased when seeds were primed with Tria. The highest metal tolerance was determined in Tria-2 (28%) as opposed to Pb-stress plants.

### 3.9. Mineral Contents Assessment

Various mineral content (Mg^+2^, Zn^+2^, K^+^, Na^+^) uptakes were assessed in both control and Pb regimes; plants grown under stress demonstrated an inhibited uptake of minerals, while those raised with Tria increased in mineral content (Table 5). Pb induces negative effects on plants for the uptake of Mg^+2^ (46%), Zn^+2^ (28%), K^+^ (48%), and Na^+^ (26%) in comparison to the control. A significant increase was observed in plant mineral uptake when pre-treated with triacontanol Tria at 20 µM compared to the control. Under control conditions, an increase of approximately 38%, 83%, 7%, and 53% was observed in Mg^+2^, Zn^+2^, K^+^, and Na^+^ uptake, respectively. Under lead conditions, Tria-2 with Pb had a maximum increase of 3.8% in Mg and 23% of Zn compared to the control, while in Tria-2 with Pb there was a decrease in K (25%) and Na (11%) uptake.

### 3.10. Total Phenol Quantification

Seeds primed with Tria plants showed more phenolic content than Pb-only seedlings. Total phenolics under Pb stress are reduced by about 18% than the control. In plants raised with Tria, the number of phenolics was increased, with the highest in Tria-2—about 74% in the control and 55% in Pb (Figure 2). Tria-1 and Tria-3 reduced the phenol content by 19% and 35% under Pb conditions over seedlings without a Pb stress environment.

### 3.11. Pearson Correlation

**Figure 3:**
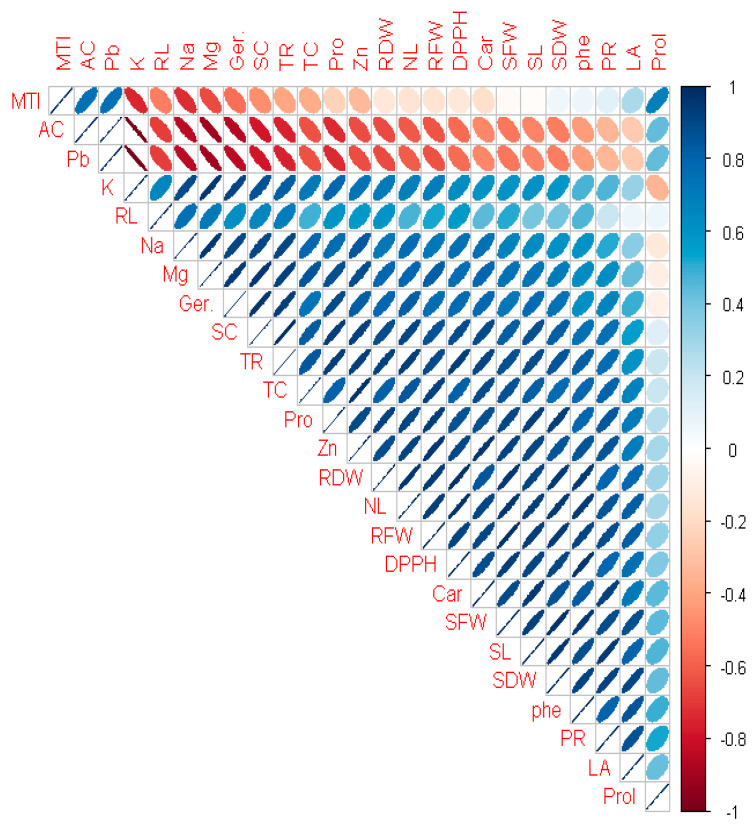
Correlation between growth and physiological parameters of *Phaseolus vulgaris* L. grown under Pb soil with the application of Tria as seed primer. Different abbreviations used in the figure are as follows: SL (shoot length), RL (root length), NL (number of leaves), LA (leaf area), Ger. (Germination percentage), SFW (shoot fresh weight), RFW (root fresh weight), SDW (shoot dry weight), RDW (root dry weight), TC (Total chlorophyll contents), Car (carotenoid contents), Mg (Magnesium uptake by plant), Zn (Zinc uptake by plant), K (Potassium uptake by plant), Na (Sodium uptake by plant), Pb (Lead uptake by plant), MTI (Metal Tolerance Index), AC (Accumulation Coefficient), TR (transpiration rate), PR (photosynthetic rate), SC (stomatal conductance), DDPH (DPPH scavenging activity), Pro (Soluble Proteins), Phe (phenolics), and Prol (Proline content).

The Pearson correlation depicts that plant Pb uptake was negative in correlation with shoot length, root length, number of leaves, leaf area, germination percentage, shoot fresh weight, root fresh weight, shoot dry weight, root dry weight, total chlorophyll contents, carotenoid content, uptake of minerals (Mg^+2^, Zn^+2^, Na^+^, K^+^), photosynthetic rate, stomatal conductance, transpiration rate, DPPH scavenging activity, total soluble proteins, and phenol and metal tolerance index. Conversely, the proline content and accumulation coefficient were found to be in positive correlation with plant Pb uptake (Figure 3).

### 3.12. Principal Component Analysis

**Figure 4:**
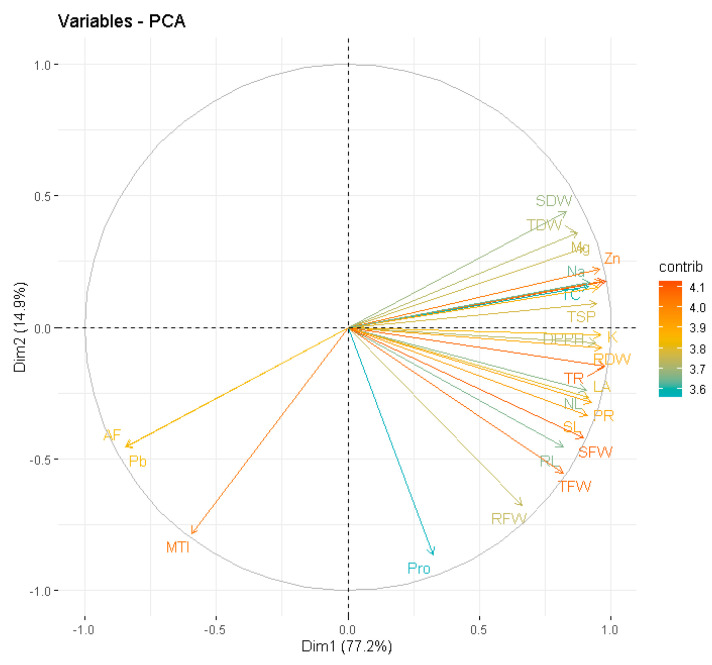
Loading plots of principal component analysis (PCA) showed a relationship between the growth and physiological parameters of *Phaseolus vulgaris* L. grown under Pb soil with the application of Tria as seed primer. Different abbreviations used in the figure are as follows: SL (shoot length), RL (root length), NL (number of leaves), LA (leaf area), SFW (shoot fresh weight), RFW (root fresh weight), TFW (Total fresh weight), SDW (shoot dry weight), RDW (root dry weight), TDW (Total dry weight), TC (Total chlorophyll contents), Mg (Magnesium uptake by plant), Zn (Zinc uptake by plant), K (Potassium uptake by plant), Na (Sodium uptake by plant), Pb (Lead uptake by plant), MTI (Metal Tolerance Index), AF (Accumulation Factor), TR (transpiration rate), PR (photosynthetic rate), DDPH (DPPH scavenging activity), TSP (Total Soluble Proteins), and Pro (Proline content).

In order to demonstrate the relationship between the physiological and growth features of *P. vulgaris* grown in soil polluted with Pb while using Tria as seed primer, the principal component analysis (PCA) loading plots were also made (Figure 4). More than 92 percent of the whole database is made up by the first two primary components, Dim1 and Dim2, which also make up the bulk of all essential components. This dataset contains 77.2 percent of Dim1 and 14.9 percent of Dim2, respectively. The first group of factors that PC1 correlates favorably are shoot length, root length, number of leaves, leaf area, germination percentage, shoot fresh weight, root fresh weight, total fresh weight, shoot dry weight, root dry weight, total dry weight, total chlorophyll contents, uptake of minerals (Mg^+2^, Zn^+2^, Na^+^, K^+^), photosynthetic rate, transpiration rate, DPPH scavenging activity, total soluble proteins, and proline. The variables that corresponded to PC1 had a significant negative correlation with the Pb concentration of plant, accumulation factor, and metal tolerance index.

## 4. Discussion

Lead (Pb) ions are transported and stored in the parts of plants growing in Pb-contaminated soils, posing a serious concern in edible crops [43]. Lead uptake from soil solutions is influenced by plant root surface area, root growth and exudates, mycorrhizal connections within the root zone, plant transpiration rates for potential absorption, and water streams. These changes influence the solubility and mobility of Pb^2+^ ions in soil solutions, as well as their potential absorption by plants [44]. When it comes to Pb absorption and translocation, different plant species have displayed diverse behaviors; in the present investigation, common bean shoots showed the maximum uptake of Pb under Pb-only regimes while Tria inhibited Pb uptake.

Plants under Pb regimes show abridged seed germination, growth, and biomass, which may be due to the reduced photosynthetic rate and reduced gaseous exchange characteristics. The decline in germination and development of plants (Table 1) is because of the adverse association of Pb with potassium, inducing abscisic acid and affecting enzymatic activities, and disrupting the membrane and the stomatal opening [7,45]. Furthermore, according to Sharma and Dubey, [46], Pb reduces biomass by interfering with metabolic activities and simulates the oxidation of indole-3-acetic acid (IAA), which is the initiative to inhibit growth; this is in accordance with current findings (Table 2). Reduced growth and biomass were observed in *Pisum sativum* [47] and *Zea mays* [48], whereas a decline in germination indexes and tolerance ability in tomato [49] under Pb stress was observed.

Seed priming with Tria enhances the germination percentage along with growth; enhanced fresh and dry biomass was also documented [50] under Pb-amended and control conditions. Triacontanol pre-treated seeds enhance α-amylase activity, which overcomes heavy metal stress. This technique also improves germination and growth of seedlings by enhancing soluble sugar and nitrogen content along with nitrate reductase performance [51]. Grain yield and quality was improved by using seed priming. Many essential components became improved, especially the antioxidant system due to improvising the production of the SOD, MDA, ascorbic acid, glutathione reductase, catalase, and specific protein release in stress conditions, such as late embryogenesis abundant (LEA), dehydrin, and aquaporin (AQP) [50,52]. Seeds primed with Tria ameliorate the Pb stress on plant growth and biomass (Table 3). Similar findings were documented in Tria-boosted physio-biochemical attributes including chlorophyll content, rate of transpiration, and stomatal conductance in wheat plants [53]. The increase in plant height by Tria might be possible by the excess formation of 9-ß-l (+)-adenosine, which positively induces plant growth [54]. Tria shows the ability to boost the metabolic and physiological responses of plants by disrupting carbohydrate metabolic enzymes [55]. A high concentration of Pb directly influences the enzymatic activity involved in photosynthesis, disrupting the chlorophyll molecule and by-producing ROS and denaturing its structure, showing a reduction in pigments that ultimately suppresses the overall growth [56]. In the current investigation, Tria (20 µmol L^−1^) shows the maximum production of photosynthetic pigments (Table 3) and carotenoids, which protects the plant from Pb toxicity by reducing the oxidative damage, leading an increment in photosynthetic activity [57]. A prominent decrease was found in photosynthetic pigments of common bean seedlings under Pb-amended soil, which is in accordance with the findings of Khalil et al. [58]. Pb uptake in plants shows a negative correlation with plant total photosynthetic contents and carotenoid contents shown in Figure 3. Leaf characteristics responsible for the enhancement in growth include CO_2_ conductance, photosynthesis, and transpirations rates, which are highly negatively influenced by Pb stress (Figure 1). Exposure to Pb also reduces the transpiration rate and damages the root system [59]. Lead toxicity caused a reduction in transpiration in soyabean [60], and cabbage, carrot, and lettuce crops [61]. Seeds primed with Tria had a significant boost in Pb tolerance in common bean by enhancing gas exchange properties in rice crop [62] and hot pepper [63] under saline conditions, which is in accordance with current findings. Triacontanol boosts the photosynthetic rate by up-regulating the expression of genes involved in the regulation of photosynthetic and rubisco activity and boosting the CO_2_ exchange rate in leaf [64,65]. Enhanced stomatal activity by foliar application of Tria was also observed in wheat grown under arsenic stress [17], and in coriander seedlings under cadmium stress [33].

Proline and Pb concentration along with Tria in plant has a positive correlation (Figure 3), as proline content is enhanced by the uptake of Pb. Proline defines the defensive strategy, functions as an osmoprotectant against heavy metals, and its aggregation indicates that the plant is under stress [66]. Proline increases the ROS scavenging activity [67], prevents the structure of chlorophyll, and also acts as a lipid membrane stabilizer [68]. Seed priming with Tria boosts the production of proline content in common beans under Pb stress conditions in control environments, and similar results were reported in soyabean [69] and *Mentha arvensis* L. [57]. The current findings revealed that seed priming with Tria not only increased the content of proline in the treatments Tria 2 + Pb and Tria 3 +Pb to eliminate those ROS, but also increased proline in Tria 2 and Tria 3 (without Pb) because Tria itself promotes the formation of osmoregulatory proline content under non-contaminated conditions. Proline is a proteogenic amino acid that accumulates both under stress and non-stress conditions as a beneficial solute in plants and plays a role in protein synthesis—mainly in cell wall proteins [70,71]. The results of the current study showed that enhanced proline levels protected the seedlings and enhanced growth (Figure 2), which is in line with other findings [72].

2,2-diphenyl-1-picrylhydrazyl (DPPH) is a potent complex secondary metabolite produced by plant in stress condition to detoxify free radicals. Its reactivity shows a decline in plants growing under abiotic stress [73,74]. Among other heavy metals, Pb shows the highest adverse effect on the antioxidant activity of plants [75]. Increments in Pb concentration in plants due to Pb present in soil show negative correlations with DDPH scavenging activity (Figure 3). Foliar application of Tria induces positive effects on DPPH activity under abiotic stress in sunflower seedlings grown in drought conditions [76], and in *Mentha arvensis* grown under nickel stress [77]. The findings of the current study demonstrated that Tria-primed seeds increase the free radical scavenging activity of common bean plants (Figure 2). Nevertheless, DPPH scavenging activity is boosted by Tria (without Pb), but not when compared to seedlings grown in Pb, indicating that Tria (Tria 1, Tria 2, and Tria 3) concentrations can modify plant DPPH activity to boost its protectants against ROS species. Triacontanol stimulates the accumulation of protein under stress environment, which creates resilience against Pb toxicity. An inverse correlation (Figure 3) was found between Pb uptake and the total soluble protein content of plant. The increase in soluble protein by Tria shows the positive impacts on plants osmotic characters, stabilizing the structure of macromolecules, and the decline in ROS, which ultimately prevents the cell death and enhanced growth [78]. The soluble protein content in common bean plant was impeded when exposed to Pb-amended soil, which is in agreement with that reported in *Sesamum indicum* [79].

Triacontanol mitigates the effect of Pb and reduces its uptake by plants, creating a high metal tolerance index (MTI), shown in Table 4; this might have an effect on the ATPases or on cation diffusion facilitators [80]. Seed primed with Tria2 showed reduced accumulation coefficient suggested reduced uptake of Pb in seedlings. Seeds primed with Tria enhance the uptake of minerals Na^+^, K^+^, and Ca^+^ by plants from soil, increase their distribution within plant tissues [81], and show a significant increment in protein content by mitigating the effect of abiotic stress. They also reduce the production of proline in hot pepper and cucumber plants [82]. Mg^+2^ uptake was also enhanced by Tria and reduced by Pb, as shown in Table 5. Mg^+2^ is essential in the structure of chloroplasts and for the formation of chlorophyll; about 2.7% of total chlorophyll weight is Mg [83]. Plant enzymes—mainly all phosphorylases and kinases—require Mg for the hydrolysis of ATP and ADP for the release of energy and phosphoric acid [84]. A decrease in the photosynthetic pigments and light absorption capacity of chloroplasts has been observed in cases of Mg deficiency [85,86].

Likewise, Zn^+2^ uptake was abridged by the Pb, causing the disruption of plant membranes, lipids, and protein, and this deficiency of Zn^+2^ lead to the perturbation of chloroplast [53]. Essential macronutrient K^+^ uptake was disturbed in Pb-stressed soil and regulated in Tria-primed plants. K^+^ plays a crucial role in the regulation and activation of various enzymes and is responsible for the osmoregulation of cells [87]. Seeds primed with Tria increased the uptake of K^+^ ions and directly enhanced their abilities to promote various plant reactions. Tria also positively regulated the uptake of micronutrient Na^+^, which was suppressed by Pb and increases the metabolic activities of plants; it also regulates the plant when K^+^ is deficient [88]. Studies reveal that Tria is significantly phyto-protectant, which increases plants’ tolerance against various heavy metals; this is in accordance with earlier research of the foliar application of Tria in wheat plants under As stress [17], and wild mint under Cd stress. Triacontanol application also improved the uptake of essential minerals N, P, and K, which were reduced by Cd stress [23].

Phenol is a secondary metabolite and its concentration in plant cells highly relies upon the cultivated soil and environmental conditions [89]. Lead toxicity reduces the phenolic content of *P. vulgaris,* depicting a negative correlation as shown in Figure 3. Nevertheless, Tria induced positive effects on the total phenolic content of common bean seedlings by alleviating Pb stress. The maize seedlings under nickel regimes [90] and coriander plants grown under Cd stress showed a reduction in phenolic content and showed positive correlations with Tria-primed seeds [33]. Overall, Tria not only enhanced common bean Pb tolerance but also enhanced the production of phenolics by suppressing Pb negative effects and improving various biochemical pathways of plants.

## 5. Conclusions

According to this study’s results, Tria as a seed-priming agent acts as a Pb alleviator for *P. vulgaris* L. growing under Pb regimes. Tria-primed seeds reduced Pb-induced phytotoxicity and improved plant development and biomass. Elevation of photosynthetic pigments, osmolyte promoter proline, and DPPH activity of plants was reported, which inhibits the noxious activity of Pb in plants. The present study advocates that at higher concentrations of Tria, there is slight reduction in uptake of minerals by plants than at lower concentrations. Tria suppressing the negative effects of Pb in plants is an effective method to enhance plant growth by reducing the use of biochemical fertilizers causing soil pollution. Further developments in promoting Tria seed-priming techniques will have beneficial impacts for seed technologists. Nevertheless, to come to a precise conclusion based on current results, a detailed molecular and proteomic analysis is necessary.

## Figures and Tables

**Table 1:** Effect of Triacontanol and Pb on Germination and Vegetative growth of *Phaseolus vulgaris* L. Seedlings.

Treatments	Growth Traits				
	Shoot Length (cm)	Root Length (cm)	No. of Leaves	Leaf Area (cm^2^)	Germination %
Control	6.12 ± 0.04 e	2.20 ± 0.07 e	4.22 ± 0.25 e	2.91 ± 0.08 e	85 ± 9.57 bc
Pb	4.07 ± 0.02 g	1.40 ± 0.04 g	2.29 ± 0.25 f	1.24 ± 0.05 g	70 ± 5.77 f
Tria 1	8.10 ± 0.04 c	2.55 ± 0.02 d	3.74 ± 0.25 b	3.82 ± 0.09 d	90 ± 5.66 b
Tria 2	10.08 ± 0.04 a	4.08 ± 0.04 b	5.23 ± 0.28 a	4.76 ± 0.18 a	95 ± 5.2 a
Tria 3	6.25 ± 0.02 de	5.45 ± 0.02 a	3.985 ± 0.25 c	2.29 ± 0.02 f	85 ± 9.57 bc
Tria 1 + Pb	6.55 ± 0.02 d	1.98 ± 0.02 c	4.20 ± 0.28 ef	3.76 ± 0.09 de	70 ± 19.14 d
Tria 2 + Pb	9.55 ± 0.02 b	2.45 ± 0.02 c	4.50 ± 0.28 bc	4.53 ± 0.14 b	85 ± 9.57 fg
Tria 3 + Pb	5.13 ± 0.04 f	1.68 ± 0.04 f	3.40 ± 0.28 d	3.92 ± 0.07 c	75 ± 9.57 e

Data exhibit means ± SE of 4 replicates. Non-identical letters specify significant difference amid the treatments at *p* ≤ 0.05. Control, Pb = 400 mg kg^− 1^ Pb, Tria-1 = 10 µmol L^−1^ of Tria, Tria-2 = 20 µmol L^−1^ of Tria, Tria-3 = 30 µmol L^−1^ of Tria.

**Table 2:** Effects of Triacontanol and Pb on Fresh and Dry Biomass of *Phaseolus vulgaris* L. Seedlings.

Treatments	Biomass Attributes			
	Shoot FW (g plant^−1^)	Root FW (g plant^−1^)	Shoot DW (g plant^−1^)	Root DW (g plant^−1^)
Control	1.25 ± 0.04 f	0.17 ± 0.04 cd	0.66 ± 0.009 e	0.06 ± 0.002 d
Pb	0.77 ± 0.08 g	0.10 ± 0.02 e	0.36 ± 0.002 f	0.03 ± 0.002 f
Tria 1	1.59 ± 0.02 c	0.20 ± 0.04 bc	0.81 ± 0.006 bc	0.09 ± 0.006 b
Tria 2	2.12 ± 0.04 a	0.26 ± 0.02 a	1.00 ± 0.005 a	0.10 ± 0.001 a
Tria 3	1.48 ± 0.04 e	0.18 ± 0.02 c	0.69 ± 0.004 c	0.08 ± 0.007 d
Tria 1 + Pb	1.50 ± 0.02 d	0.19 ± 0.06 c	0.70 ± 0.003 c	0.07 ± 0.004 c
Tria 2 + Pb	1.78 ± 0.04 b	0.21 ± 0.02 b	0.97 ± 0.001 b	0.09 ± 0.004 b
Tria 3 + Pb	1.37 ± 0.04 e	0.15 ± 0.02 d	0.67 ± 0.007 d	0.06 ± 0.008 de

Data exhibit means ± SE of 4 replicates. Non-identical letters specify significant difference amid the treatments at *p* ≤ 0.05. Control, Pb = 400 mg kg^− 1^ Pb, Tria-1 = 10 µmol L^−1^ of Tria, Tria-2 = 20 µmol L^−1^ of Tria, Tria-3 = 30 µmol L^−1^ of Tria.

**Table 3:** Effects of Triacontanol and Pb on Photosynthetic Pigments of *Phaseolus vulgaris* L. Seedlings.

Treatments	Photosynthetic Pigments			
	Chl *a* (mg g^−1^ FW)	Chl *b* (mg g^−1^ FW)	Total Chlorophyll (mg g^−1^ FW)	Carotenoids (mg g^−1^ FW)
Control	0.90 ± 0.05 d	0.69 ± 0.15 e	0.38 ± 0.11 d	0.72 ± 0.002 f
Pb	0.69 ± 0.05 e	0.58 ± 0.14 f	0.27 ± 0.12 f	0.66 ± 0.10 h
Tria 1	1.14 ± 0.10 bc	0.98 ± 0.18 bc	0.46 ± 0.16 ab	0.88 ± 0.003 c
Tria 2	1.46 ± 0.01 a	1.29 ± 0.16 a	0.62 ± 0.09 a	1.14 ± 0.01 a
Tria 3	1.00 ± 0.06 b	0.83 ± 0.02 ab	0.38 ± 0.03 d	0.76 ± 0.09 d
Tria 1 + Pb	1.13 ± 0.09 bc	0.94 ± 0.17 c	0.43 ± 0.16 b	0.75 ± 0.008 e
Tria 2 + Pb	1.31 ± 0.13 b	1.08 ± 0.12 b	0.39 ± 0.14 c	0.93 ± 0.02 b
Tria 3 + Pb	0.91 ± 0.06 c	0.72 ± 0.04 d	0.31 ± 0.03 e	0.69 ± 0.01 g

Data exhibit means ± SE of 4 replicates. Non-identical letters specify significant difference amid the treatments at *p* ≤ 0.05. Control, Pb = 400 mg kg^− 1^ Pb, Tria-1 = 10 µmol L^−1^ of Tria, Tria-2 = 20 µmol L^−1^ of Tria, Tria-3 = 30 µmol L^−1^ of Tria.

**Table 4:** Impact of Pb and Triacontanol on the Uptake, Accumulation, and Tolerance Index of Pb on *Phaseolus vulgaris* L.

Treatments	Pb Uptake by Plant (mg g^−1^ DW)	Accumulation Coefficient (AC)	Metal Tolerance Index (MTI)%
Control	-	-	-
Lead	0.17 ± 0.006 a	0.58 ± 0.02 a	54.81 ± 1.11 d
Tria 1	-	-	-
Tria 2	-	-	-
Tria 3	-	-	-
Tria 1 + Pb	0.11 ± 0.0006 c	0.38 ± 0.002 c	107.12 ± 1.4 b
Tria 2 + Pb	0.10 ± 0.001 d	0.34 ± 0.005 d	147.02 ± 2.03 a
Tria 3 + Pb	0.12 ± 0.0005 b	0.41 ± 0.001 b	101.19 ± 1.21 c

Data exhibit means ± SE of 4 replicates. Non-identical letters specify significant difference amid the treatments at *p* ≤ 0.05. Control, Pb = 400 mg kg^− 1^ Pb, Tria-1 = 10 µmol L^−1^ of Tria, Tria-2 = 20 µmol L^−1^ of Tria, Tria-3 = 30 µmol L^−1^ of Tria. (-) indicates there is no detection of Pb.

**Table 5:** Impact of Pb Triacontanol on the Uptake of Mineral Contents of *Phaseolus vulgaris* L.

Treatments	Mg^+2^ (mg g^−1^ DW)	Zn^+2^ (mg g^−1^ DW)	K^+^ (mg g^−1^ DW)	Na^+^ (mg g^−1^ DW)
Control	10.45 ± 0.43 e	0.42 ± 0.02 e	39.60 ± 0.10 c	30.11 ± 0.18 d
Pb	05.60 ± 0.55 g	0.30 ± 0.05 g	20.42 ± 0.13 g	22.02 ± 0.33 g
Tria 1	11.65 ± 0.25 b	0.56 ± 0.11 b	40.51 ± 0.13 b	38.01 ± 0.43 f
Tria 2	14.49 ± 0.56 a	0.77 ± 0.14 a	42.40 ± 0.19 a	46.21 ± 0.42 a
Tria 3	11.15 ± 0.39 c	0.48 ± 0.04 cd	37.80 ± 0.09 d	37.12 ± 0.36 c
Tria 1 + Pb	07.37 ± 0.26 b	0.46 ± 0.02 d	25.39 ± 0.09 f	22.21 ± 0.33 g
Tria 2 + Pb	10.85 ± 0.55 d	0.52 ± 0.01 c	29.59 ± 2.05 e	26.66 ± 0.25 e
Tria 3 + Pb	06.27 ± 0.30 f	0.36 ± 0.04 f	24.00 ± 0.39 f	24.88 ± 0.16 f

Data exhibit means ± SE of 4 replicates. Non-identical letters specify significant difference amid the treatments at *p* ≤ 0.05. Control, Pb = 400 mg kg^− 1^ Pb, Tria-1 = 10 µmol L^−1^ of Tria, Tria-2 = 20 µmol L^−1^ of Tria, Tria-3 = 30 µmol L^−1^ of Tria.

## Data Availability

The original contributions presented in the study are included in the article, further inquiries can be directed to the corresponding author/s.

## Supplementary Materials

The following supporting information can be downloaded at: https://www.mdpi.com/article/10.3390/plants12081672/s1, Figure S1: Effect of Triacontanol and Pb on Germination and Morpho-physiological Characteristics of *Phaseolus vulgaris* L. Seedlings.
