# Supplementary material for: Seed Priming with Triacontanol Alleviates Lead Stress in Phaseolus vulgaris L. (Common Bean) through Improving Nutritional Orchestration and Morpho-Physiological Characteristics
Source: Plants (Basel). 2023 Apr 17;12(8):1672. doi: 10.3390/plants12081672 (PMC10145083; doi:10.3390/plants12081672)
Supplement: Supplementary file 1 [file plants-12-01672-s001.zip › plants-2249001-supplementary.pdf]

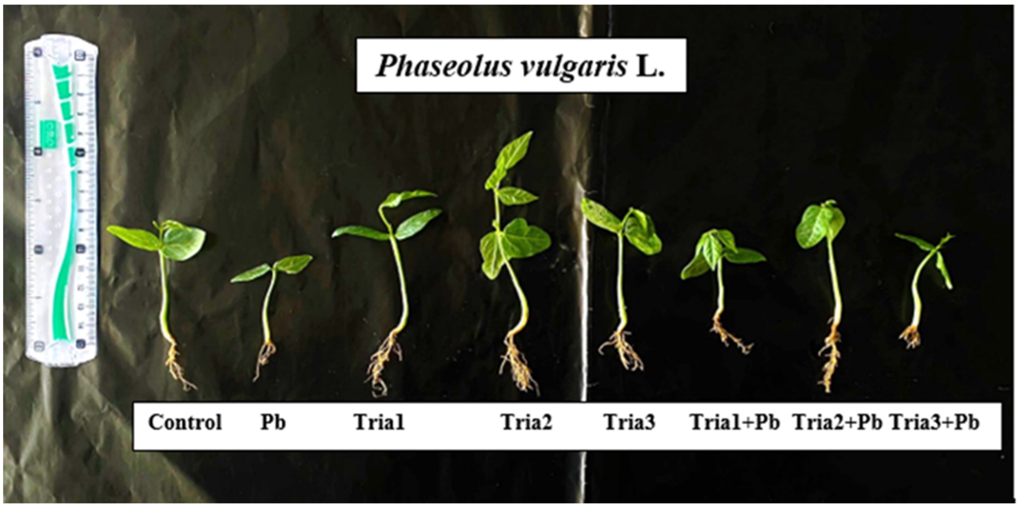

Figure S1. Effect of Triacontanol and Pb on Germination and Morpho-physiological Characteristics of *Phaseolus vulgaris* L. Seedlings. Control=uncontaminated control, Pb = 400mg kg^{-1} Pb, Tria-1 =10µM, Tria-2 =20 µM, Tria-3 =30µM of Tria.
